# Endomicroscopic and Transcriptomic Analysis of Impaired Barrier Function and Malabsorption in Environmental Enteropathy

**DOI:** 10.1371/journal.pntd.0004600

**Published:** 2016-04-06

**Authors:** Paul Kelly, Ellen Besa, Kanekwa Zyambo, John Louis-Auguste, James Lees, Themba Banda, Rose Soko, Rosemary Banda, Beatrice Amadi, Alastair Watson

**Affiliations:** 1 Blizard Institute, Barts and The London School of Medicine, Queen Mary University of London, London, United Kingdom; 2 Tropical Gastroenterology and Nutrition group, University of Zambia School of Medicine, Lusaka, Zambia; 3 Norwich Medical School, University of East Anglia, Norwich, United Kingdom; University of Virginia Health System, UNITED STATES

## Abstract

**Introduction:**

Environmental enteropathy (EE) is associated with growth failure, micronutrient malabsorption and impaired responses to oral vaccines. We set out to define cellular mechanisms of impaired barrier function in EE and explore protective mechanisms.

**Methods:**

We studied 49 adults with environmental enteropathy in Lusaka, Zambia using confocal laser endomicroscopy (CLE); histology, immunohistochemistry and mRNA sequencing of small intestinal biopsies; and correlated these with plasma lipopolysaccharide (LPS) and a zinc uptake test.

**Results:**

CLE images (median 134 for each study) showed virtually ubiquitous small intestinal damage. Epithelial defects, imaged by histology and claudin 4 immunostaining, were predominantly seen at the tips of villi and corresponded with leakage imaged *in vivo* by CLE. In multivariate analysis, circulating log-transformed LPS was correlated with cell shedding events (β = 0.83; *P* = 0.035) and with serum glucagon-like peptide-2 (β = -0.13; *P* = 0.007). Zinc uptake from a test dose of 25mg was attenuated in 30/47 (64%) individuals and in multivariate analysis was reduced by HIV, but positively correlated with GLP-2 (β = 2.72; *P* = 0.03). There was a U-shaped relationship between circulating LPS and villus surface area. Transcriptomic analysis identified 23 differentially expressed genes in severe enteropathy, including protective peptides and proteins.

**Conclusions:**

Confocal endomicroscopy, claudin 4 immunostaining and histology identify epithelial defects which are probably sites of bacterial translocation, in the presence of which increased epithelial surface area increases the burden of translocation. GLP 2 and other protective peptides may play an important role in mucosal protection in EE.

## Author Summary

Environmental enteropathy is a widespread problem in adults and children in many disadvantaged populations, particularly in the tropics. It is not attributable to one specific infectious agent, but likely due to several insults of environmental origin. It is associated with growth failure in children, impaired responses to oral vaccines, and contributes to micronutrient deficiencies. Many of these problems can be related to immune activation, but the pathways of immune activation are unknown. Here we show that translocation of gut microbes and their molecular components is associated with defects in the epithelial lining of the gut, imaged using advanced endoscopic techniques. We also report evidence that these defects are associated with failure of endocrine and paracrine repair mechanisms which in health should restore the integrity of the intestinal barrier. These observations may open up new approaches for therapy for a neglected tropical disorder which impairs the health of millions of adults and children.

## Introduction

Environmental enteropathy (EE) or environmental enteric dysfunction (EED) is an asymptomatic disorder which was originally described as ‘tropical enteropathy’ [1,2]. First recognised as an asymptomatic variant in small intestinal mucosal architecture [3], then as a cause of subtle malabsorption without obvious clinical consequences, it is now recognised as a major contributor to the poor linear growth (stunting) of millions of children in many of the world’s most disadvantaged populations [4]. Stunting affects 40% of Zambian children under 5 years of age [5] and is an independent predictor of mortality, morbidity in later life, and lifelong economic disadvantage [4]. It seems likely that adverse environmental conditions (poor sanitation most prominently [6]) lead to recurrent intestinal damage causing microbial translocation and systemic inflammation [7]. This damage is associated with impaired responses to oral vaccines such as polio, cholera and rotavirus [7,8].

In studies in The Gambia, linear growth velocity during infancy was inversely associated with intestinal permeability, as reflected in increased lactulose permeability relative to mannitol, and with serum antibodies to lipopolysaccharide [9]. This is the most direct evidence that microbial translocation is important in the process of stunting. The mechanisms by which microbial translocation causes stunting are not well defined, but probably the stimulation of innate immune cells by ligands for toll-like receptors such as TLR4 and TLR5 lead to secretion of pro-inflammatory molecules [10,11] which drive anorexia and disordered partitioning of nutrients. There is abundant evidence that microbial translocation with resultant systemic inflammation contributes to the pathogenesis of other diseases such as cirrhosis [12,13], HIV [10,14,15], non-alcoholic fatty liver disease [16], Crohn’s disease [10,17] and coeliac disease [10], and microbial translocation predicts post-operative sepsis [18], so further elucidation of these pathways is of considerable importance. EE itself is probably initiated by clinical [19] and sub-clinical [20] infections and changes in the microbiota.

The intestinal barrier is a surface which divides host and environment in the gut; although poorly defined currently, it includes the mucus layer, secreted antimicrobial peptides and IgA, epithelial cells and innate and adaptive immune cells. Epithelial cell polarity, and the viability of the monolayer, are dependent on the formation of tight junctions, adherens junctions, and desmosomes [21]. Tight junctions form a key part of the epithelial barrier, and a key point at which ion selectivity is regulated by some of the 26 human claudin genes [22]. Cellular defects, such as those due to epithelial cell damage by TNF, cause major impairment of barrier function [23], but there has been minimal work on their role in bacterial translocation. Disturbances of digestion and absorption are well described in EE and EED, but the long-term implications of microbial translocation in adults in tropical and economically disadvantaged populations remain almost unexplored.

Confocal laser endomicroscopy (CLE) is a new technique for the visualisation and quantification of intestinal epithelial barrier integrity [24]. It is the only technique that can visualise epithelial cell shedding and sites of barrier loss in patients during endoscopy in real time. Precise anatomic sites of barrier loss can be identified at a cellular level as “plumes” of fluorescein effluxing from the mucosal epithelial surface into the lumen [25]. CLE has been used to demonstrate that excessive cell shedding, epithelial microerosions and barrier loss predict relapse of IBD over a 12 month period [25]. These data emphasise the importance of barrier integrity in the pathogenesis of intestinal inflammation.

The role of epithelial damage and barrier loss in the pathways of microbial translocation and malabsorption in EE has not, to our knowledge, been studied. As CLE cannot currently be carried out in children due to the size of the instrument tip, we studied the well-established enteropathy seen in adults in Lusaka [20]. We set out to identify the extent to which cellular defects contribute to these pathophysiological processes. We studied volunteers from an impoverished urban African community, and correlated histopathological lesions, tight junction protein expression (claudin 4), and *in vivo* imaging using confocal endomicroscopy with plasma markers of translocation and zinc uptake as a marker of micronutrient absorptive capacity. As hypochlorhydria may contribute to intestinal colonisation [26] we measured gastric pH, and we measured serum intestinal type fatty acid binding protein (i-FABP) as a marker of epithelial damage, inflammatory markers, and glucagon-like peptide-2 (GLP-2) in blood. Finally, transcriptomic analysis was used to search for novel determinants of barrier function.

## Methods

### Ethics statement

Approval for the study was obtained from the University of Zambia Biomedical Research Ethics Committee (006-01-13, 11^th^ April, 2013).

### Participants and recruitment

Misisi compound is a district of southern Lusaka in which we have been conducting studies of EE since 1999 [20]. Adult volunteers were recruited using a 3-stage consent process which involves door-to-door invitations, focus group discussions and individual interviews leading to informed written consent, and recently we have incorporated laboratory visits for interested potential participants [27]. Participants with concurrent illness, pregnancy, or use of antibiotics or NSAIDs within one month, or recent helminth infection detected in a single stool sample, were excluded from study. During recruitment, a thorough clinical assessment was carried out, including simple anthropometry (weight, height, mid upper arm circumference).

### Clinical evaluation

Following an overnight fast, blood and urine samples were collected and then 100ml of a test solution containing 25mg zinc as zinc sulphate heptahydrate (Sigma, Poole, UK) was taken orally. After 3 hours, a further blood sample was collected. All samples were centrifuged at 537*g* for 15 minutes. Endoscopy was performed the next day, under conscious sedation (2.5-10mg diazepam, 50-100mg pethidine intravenously), using a Pentax EG3870CIK confocal laser endomicroscope. A sample of gastric fluid (2ml) was aspirated from the fundus of the stomach for pH measurement using pH test strips (Sigma, Poole, UK). Once a stable position was obtained in the second part of the duodenum, as gently as possible so as to avoid trauma to the mucosa, 5-10ml of 2% fluorescein was given intravenously at time 0 and images collected continuously for 10 mins. Three biopsies were collected into saline, orientated under a binocular microscope, then fixed in formal saline. Four biopsies were snap-frozen in liquid nitrogen for RNA analysis. HIV serological testing was carried out with consent in all cases.

### Image processing

During the 10 minute image collection period, between 100 and 400 images were collected for later analysis (for details see S1 Text), while slowly moving the instrument around the second and third parts of the duodenum. We determined the Watson score, a scoring system for assessing epithelial integrity in IBD [24].

### Morphometry

Morphometry was performed on haematoxylin and eosin (H&E) stained biopsy sections (Fig A in S1 Text). Measurements were only made where crypts could be seen to have been sectioned along their entire length [20]. Villous height, crypt depth, epithelial surface area (represented by villous perimeter per unit length of mucosa) and villous unit volume (represented by villous area per unit length of mucosa) were measured by a single observer (JLA) with no access to other data, using the NanoZoomer digital pathology system (Hamamatsu Photonics, Shizuoka, Japan). Epithelial defects were evaluated semi-quantitatively (none, few, many); in a subset of 14 (seven mild enteropathy, seven severe) biopsies the defects were fully quantified to confirm the validity of the ordinal scoring (*P*<0.05).

### Claudin 4 imaging

Claudin-4 immunostaining was performed using standard techniques (for details, see S1 Text).

### Zinc uptake test

Plasma samples before and after 25mg oral zinc (as zinc sulphate heptahydrate) were obtained from blood collected into trace element-free lithium heparin tubes. Samples were analysed in an Optima ICP Plasma Spectrometer (Perkin-Elmer, Midrand, South Africa) at 206.2 nm. Reference values for the zinc ‘tolerance’ (uptake) test were obtained from Valberg et al [28] which indicates a lower 95% confidence limit of 9.5 μmol/l for the increment in plasma zinc at 3 hours in healthy Canadian volunteers.

### Biomarkers of translocation, epithelial integrity and inflammation

Biomarkers of microbial translocation and inflammation (LPS, LPS binding protein, CRP, sCD14, CD163, GLP-2 and α1-antitrypsin) were compared with confocal images and zinc uptake in order to explore the barrier defects further and to identify potential biomarkers for use in further research. All samples for analysis were collected immediately prior to endoscopy, in the fasted state.

### Transcriptome analysis

Biopsies were chosen to represent the extreme ends of the spectrum of enteropathy as assessed by CLE, including four with no plumes and four with plumes seen in >35% of images. For further details of sequencing and RKPM/NOIseq [29] analysis see S1 Text.

### Data analysis

Data analysis was carried out using Stata 13 (Stata Corp, College Station, TX). All the CLE and translocation variables were treated as continuous variables and determined to be non-normally distributed using the Shapiro-Wilk test (*P*<0.0001 in all cases). Spearman’s rank correlation coefficient and the Kruskal-Wallis test were used for hypothesis testing, and correlation analysis used log-transformed LPS concentrations. Linear regression was used on continuous variables and unconditional logistic regression was used on binary variables to search for correlates of barrier failure. Multiple linear regression modelling of LPS and plasma zinc increment (in separate models) was carried out using an automatic backwards stepwise command in Stata 13 with all CLE variables, GLP-2, Lactulose recovery alone and as a ratio with rhamnose, FABP, gastric pH, HIV status, and all the variables in Table 1. Scatter plots of LPS against morphometric measures suggested a U-shaped relationship, so fractional polynomial regression was used to model translocation against them.

**Table 1:** Clinical characteristics of study participants.

	HIV seronegative (n = 35)	HIV seropositive (n = 14)	*P*
Sex (M:F)	15:20	3:11	0.20
Age (years): median (IQR) [range]	24 (21–37) [18–55]	36 (31–47) [22–55]	0.008
Secondary education (n, %)	18 (52)	7 (50)	1.00
Asset scores	2 (1–4) [0–4]	3 (2–3) [0–4]	0.97
Household hygiene score	6 (5–8) [4–10)	6 (5–8) [4–9)	0.82
Smoking, ever	2 (6)	2 (14)	0.57
Alcohol, ever consumed	14 (42)	5 (30)	1.00
Ever boil drinking water	7 (20)	1 (7)	0.41
Ever chlorinate drinking water	20 (57)	11 (79)	0.20
BMI (kg/m^2^; median, IQR)	22.4 (20.2–24.9) [17.0–52.0]	23.4 (20.7–30.3) [16.9–44.3]	0.40
MUAC (cm; median, IQR)	26.4 (25.0–29.0) [21.8–34]	27.5 (25.5–34) [20.4–43]	0.25
Taking ART (n, %)		6 (43)	
CD4 count (cells/μl)			
on ART (n = 6)		513 (336–699) [160–883]	0.67
not on ART (n = 8)		520 (369–690) [218–816]	

Values shown are median (interquartile range), and range in square brackets. ART, anti-retroviral therapy. The asset score is based on ownership of the house, electricity in the house, radio, and mobile phone. The household hygiene score is generated on a scale of 1−10, with up to two points being given for each of: overall cleanliness of the house, water storage facilities, food storage facilities, handwashing facilities and their use, and sanitation facilities

## Results

Of 81 adults who volunteered for the study, 61 adults were recruited and 20 were excluded (withdrawals, helminth infections, recent diarrhoea or antibiotic or NSAID use). CLE images were not available in 12 participants (one pyloric stenosis, one failure of sedation, ten technical laser problems), so we report findings in 49 adults. HIV seropositive and seronegative adults differed in age, but not sex, nutritional status, smoking or drinking habits, household living conditions, or treatment of drinking water (Table 1). The median CD4 count in 14 HIV positive participants was >500 cells/μl in both those receiving standard anti-retroviral therapy (ART) and those who were ART-naive, with only 1 participant having a CD4 count below 200 cells/μl and 7 below 350 cells/μl. No adverse events were noted during this study, apart from two instances of pain at the site of venous cannulation.

### Confocal endomicroscopy

**Fig 1:**
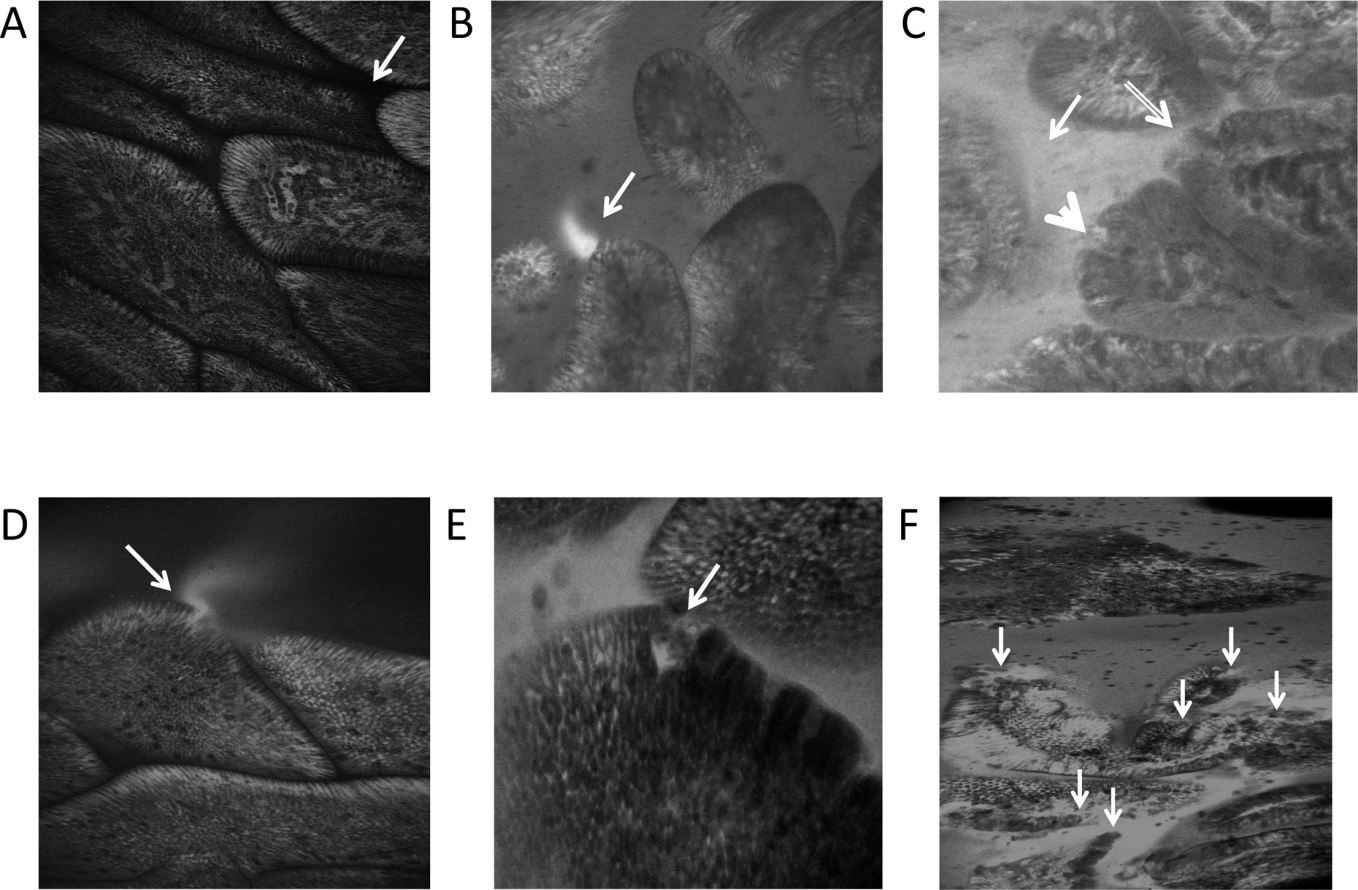
**Confocal laser endomicroscopy images of (A) normal villous epithelium showing black, fluorescein-free lumen (arrow); (B) a plume of fluorescein (arrow) seen against a dark lumen; (C) fluorescein filling the lumen (thin arrow), a microerosion (thick arrow) and a breach at a villus tip (hollow arrow); (D) single cell defect with fluorescein plume (arrow), (E) example of a multiple cell defect, a small microerosion where two cells are seen to have detached from the basement membrane (arrow), and (F) total breakdown of epithelial contiguity (arrows).**

Image quality was unsatisfactory in 8 image sets, so CLE data are presented on 41 participants. Examples of normal epithelium with an intact barrier, fluorescein leakage “plumes”, single cell epithelial defects and epithelial erosions are presented in Fig 1.

There were only 8 participants (20%) in whom no luminal leakage of fluorescein was detected in the lumen, and only 2 (5%) with no fluorescein plumes. The high frequency with which these lesions was observed contrasts sharply with previous work in a healthy German population, using the same model of Pentax CLE instrument, in which eight controls had a Watson score of 1 (i.e. no fluorescein plumes / leakage), two scored 2 (i.e. fluorescein leakage but no microerosions) and none scored 3 (fluorescein leakage with any microerosions) [30]. In our participants, 4 (10%) had a Watson score of 1, 6 (15%) scored 2, and 31 (76%) had a Watson score of 3 (*P*<0.0001 for the difference between Zambian and German scores). All the confocal endomicroscopy appearances were very highly correlated with each other (Table A in S1 Text). The identification of plumes always led to detectable luminal fluorescein, but the converse was not true, suggesting that when the rate of leakage is high the luminal fluorescein obscures plumes which are only visible against a dark lumen background. While erosions and single cell defects were invariably associated with fluorescein leakage into the lumen, a large proportion of leakage occurred in the absence of visible epithelial lesions in the optical sections obtained at CLE (Fig B in S1 Text). This is likely to be due to epithelial lesions outside the optical plane of the CLE images, but may also be due to disorganised tight junction proteins even in the absence of a break in the epithelium. Of the biomarkers and risk factors listed in Table 2, only FABP was correlated with plumes (Fig C in S1 Text).

**Table 2:** Biomarkers of translocation and intestinal integrity.

	HIV seronegative (n = 35)	HIV seropositive (n = 14)	*P*
*Measures of translocation*			
LPS (EU/ml) (median, IQR)	223 (99–397)	176 (102–286)	0.50
LPS binding protein (ng/ml)	22.9 (19.8–29.0)	29.2 (26.6–35.8)	0.01
C-reactive protein (mg/l)	0.95 (0.47–3.2)	7.52 (1.6–12.9)	0.01
soluble CD14 (ng/ml)	1.62 (1.34–1.87)	1.74 (1.46–2.10)	0.30
CD163 (ng/ml)	618 (408–797)	599 (450–855)	0.71
*Morphometry and epithelial defects*			
Villus height (μm)	219 (196–248) n = 25	215 (204–269) n = 8	0.48
Crypt depth (μm)	161 (145–172)	168 (131–179)	0.93
Villus height: crypt depth ratio	1.35 (1.25–1.50)	1.48 (1.30–1.61)	0.21
Epithelial surface area	655 (534–716)	486 (460–548)	0.02
SA:volume ratio	0.032 (0.027–0.035)	0.028 (0.025–0.032)	0.15
Epithelial defects (H&E):			
none	1	0	0.82
few	12	4	
many	24	11	
*Defects imaged using claudin 4 immunostaining*			
Villus tip lesions present	10/27 (34%)	1/9 (11%)	0.22
Epithelial defects present	16/27 (59%)	5/9 (56%)	1.00
*Zinc uptake*			
Baseline zinc concentration (μmol/l)	11.9 (9.0–15.0)	10.4 (6.8–15.2)	0.24
Rise in plasma zinc concentration over 3 hours (μmol/l)	7.2 (5.2–12.7)	5.4 (2.5–7.6)	0.07
*Potential predictors of translocation and malabsorption*			
Gastric pH	1.5 (1.5–2.5)	5.5 (3.0–5.5)	0.002
Fatty Acid Binding Protein (pg/ml)	0.59 (0.45–0.86)	0.66 (0.49–1.40)	0.63
GLP-2 (ng/ml)	1.2 (0.8–2.0)	0.6 (0–1.9)	0.12
Duodenal α_1_-antitrypsin (WB):			
none	17	4	0.10
weak	7	2	
moderate	5	3	
strong	6	5	
Duodenal MBL (WB):			
none	13	5	0.96
weak	7	4	
moderate	8	4	
strong	12	7	
Faecal α_1_-antitrypsin (μg/ml)	0.10 (0.02–0.26)	0.21 (0.04–0.36)	0.38

LPS, lipopolysaccharide; EU, endotoxin units; CD, cluster of differentiation marker; WB, Western blot; MBL, mannose binding lectin. WB data are expressed as an ordinal scale (none, weak, moderate, strong intensity of bands). Statistical testing used the Kruskal-Wallis test for continuous variables or the χ^2^ test for trend for ordered categorical variables. Morphometric data show much reduced villus height and villus:crypt ratio compared with UK normal values (Table B in S1 Text).

### Morphometry

**Fig 2:**
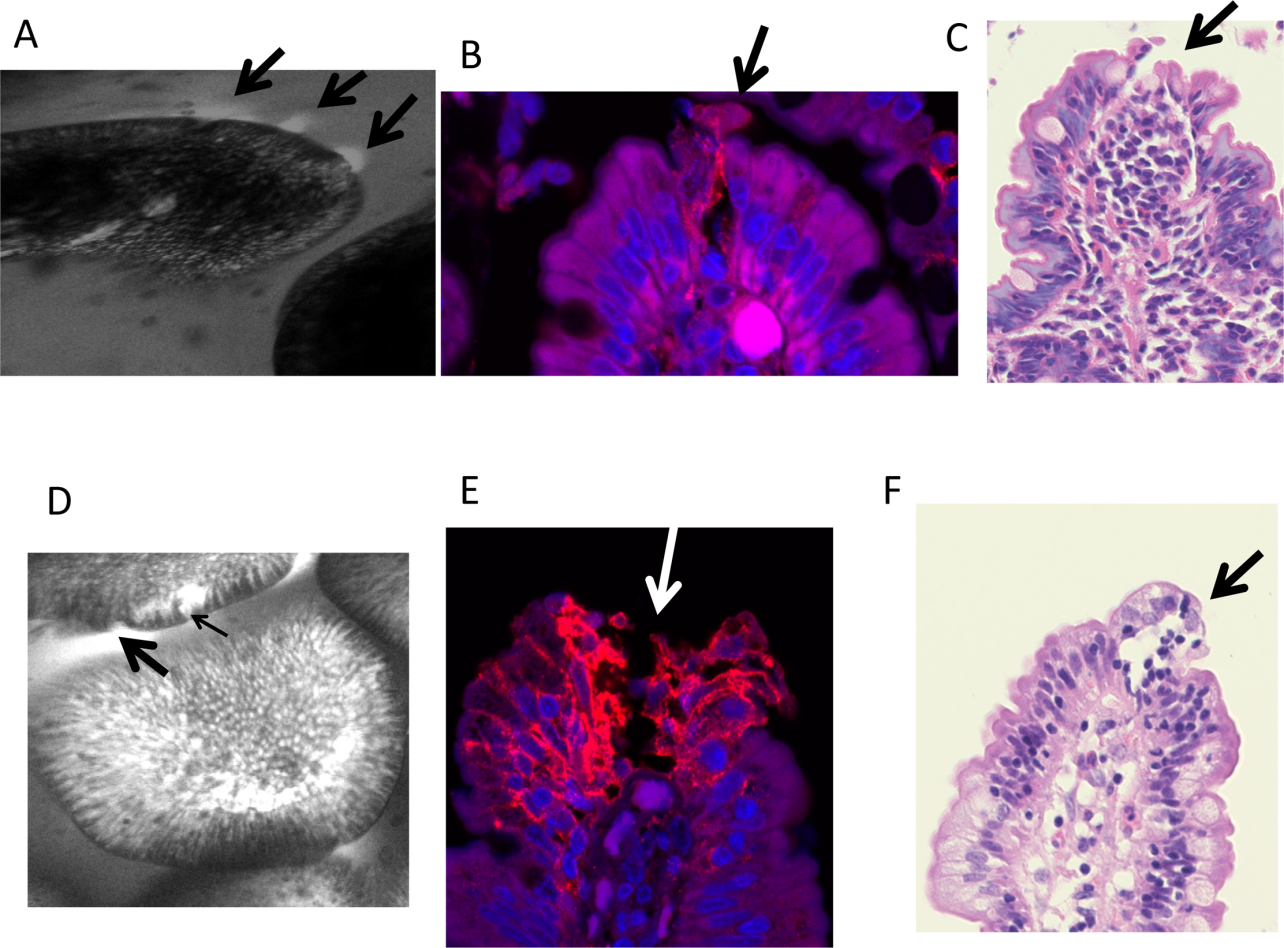
**Corresponding confocal laser endomicroscopy images (A, D), claudin 4 immunostains (B, E) and histology (C, F) from two individuals (A-C and D-F)**. In A, three plumes (arrows) are seen; in B claudin 4 immunostaining is disorganised at the site of cell shedding, and in C an epithelial defect is seen with a breach of the basement membrane (arrow). In D, a microerosion (thin arrow) and plume (thick arrow) are associated; in E claudin 4 immunoreactivity is disorganised along track of cell loss at a villus tip (arrow) with exposure of the basement membrane; and the corresponding histology image is shown in F.

Villous blunting and crypt lengthening were seen in all biopsies, and no biopsies had a Villus Height:Crypt Depth ratio of greater than 2.2:1 (Table 2) compared to a normal ratio of 3:1 or more [31]. All biopsies showed lamina propria inflammation. No significant differences were seen between HIV infected and uninfected individuals’ biopsies, with the exception of surface area (represented by villus perimeter). Villus height and crypt depth were positively correlated (ρ = 0.49; *P* = 0.01). Epithelial defects were common (Fig 2 and Table 2). Some of the defects seen by histology were surprisingly large (Fig 2C and Fig D in S1 Text), but comparison with confocal images obtained *in vivo* suggested that these defects were present before the biopsy was collected (Fig 2A and 2D, and Fig D in S1 Text).

### Immunohistochemical analysis of claudin 4 expression

**Fig 3:**
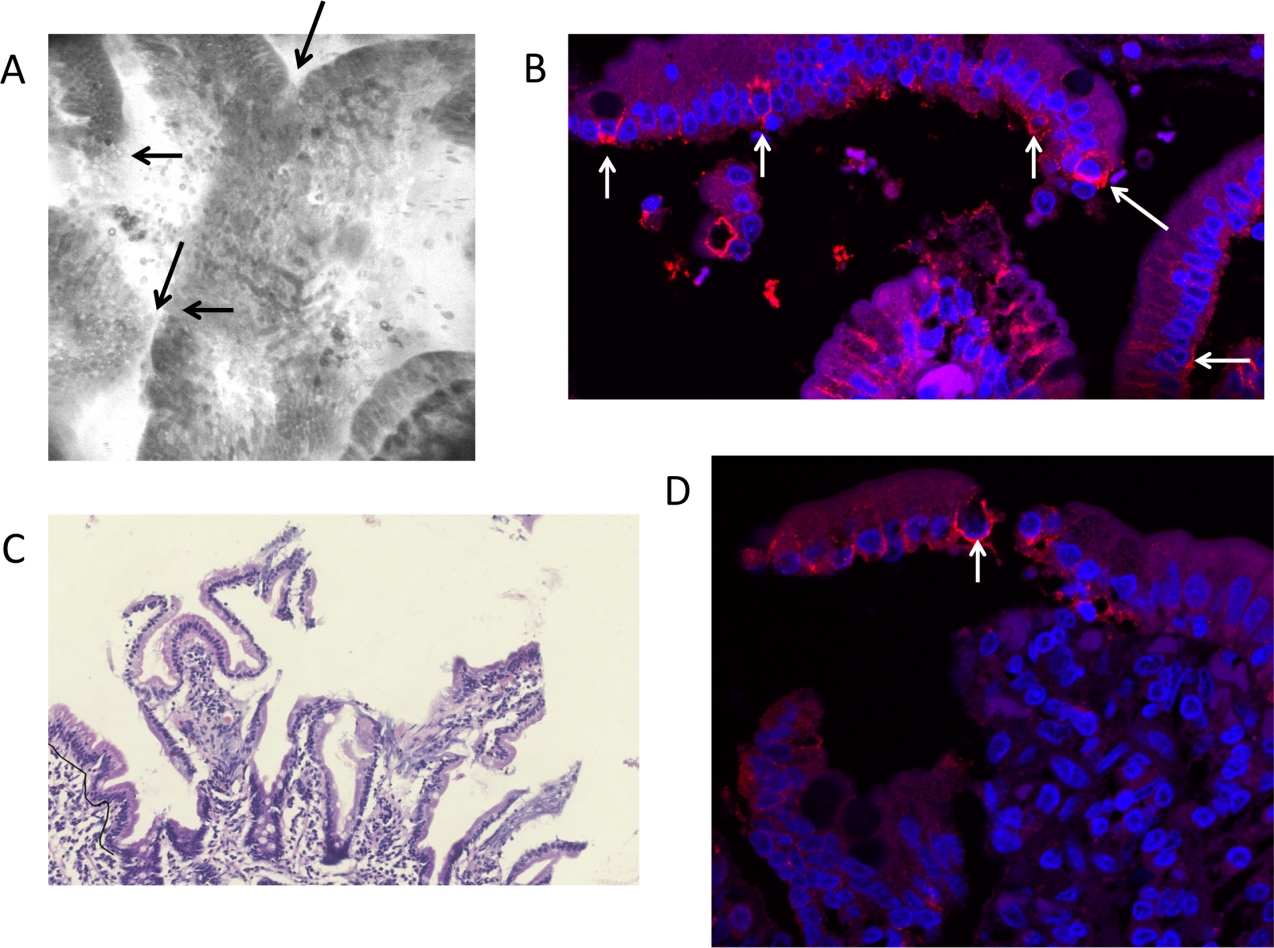
**A-D Four images from the same participant showing complete loss of epithelial integrity even though the mucosa appeared normal at endoscopy**. In A, a loss of epithelial continuity is apparent at several points (arrows) in a CLE image. In B, funnels of claudin 4 are visible (arrows) at the point of cell shedding from the epithelium [32], but non-detaching epithelium displays less immunoreactivity. In C, complete epithelial detachment is apparent. In D, increased claudin 4 immunoreactivity is seen in the detaching epithelium compared to the intact epithelium, with at least one prominent funnel (arrow).

Having identified epithelial defects using CLE, and having identified that some leakage occurred in the absence of detectable epithelial defects, we then analysed tight junction integrity and distribution using staining of claudin 4, a tight junction protein [21,22]. We have previously demonstrated that tight junction proteins redistribute in “funnels” around shedding cells to seal the epithelium at sites of cell shedding [32,22]. As with other tight junction proteins previously studied, claudin 4 funnels were detected at sites of cell shedding and in epithelial sheets that appeared to have recently detached from villus epithelium (Fig 3 and Fig D in S1 Text). Where more than one adjacent cell is shed the redistribution of claudin-4 and other tight junction proteins are no longer able to seal the epithelial monolayer (Fig 2E and Fig D in S1 Text). There was no difference in expression between HIV infected and uninfected tissue.

### Bacterial translocation

**Fig 4:**
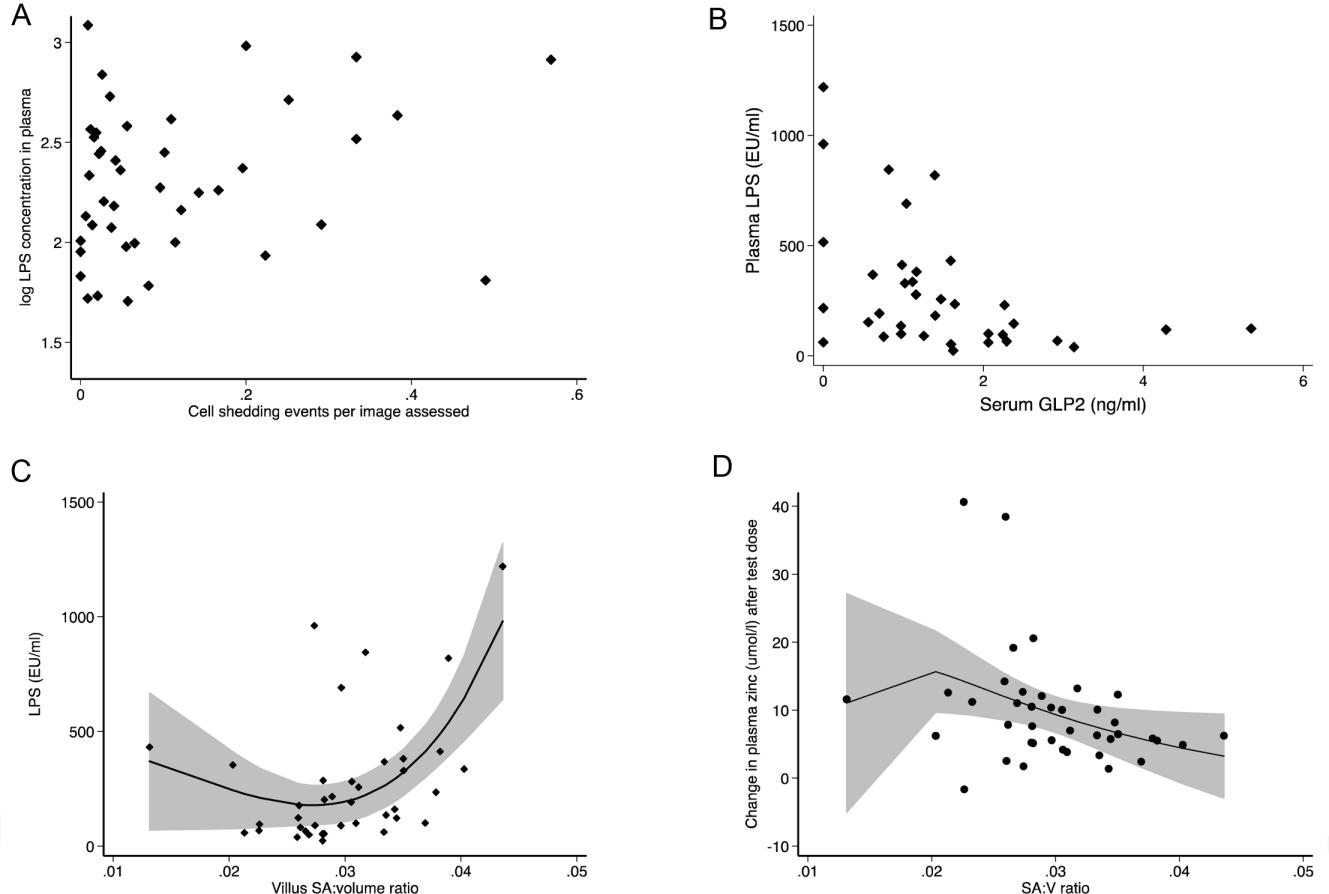
**Lipopolysaccharide concentrations (Endotoxin Units/ml) in plasma were correlated with: (A) cell shedding (β = 0.83; *P* = 0.035 in the multivariate model); (B) GLP-2 (β = -0.13; *P* = 0.007 in the multivariate model); and (C) villus surface area:volume ratio estimated from morphometry of intestinal biopsies**. In (C) the data points are shown together with the curve and 95% confidence limits from a fractional polynomial regression model which was significant with 2 degrees (*P* = 0.007 and *P* = 0.025). (D) Polynomial fractional regression plot as in (C) but showing the relationship between zinc uptake and surface area:volume ratio.

Plasma LPS was measured to gauge bacterial translocation (Table 2). LPS was detectable in all samples; it was unaffected by HIV status, whereas two of the indirect markers (LBP and CRP) were higher in HIV seropositive participants (Table 2). Regression models were constructed including all the CLE measurements together with gastric pH, FABP, and GLP-2, but without morphometric measurements. In the regression models of log-transformed LPS, cell shedding (β = 0.83, n = 43; *P* = 0.035) and GLP-2 (β = -0.13, n = 43; *P* = 0.007) were associated with LPS in the final model (univariate scatter plots are shown in Fig 4). Thus, low circulating GLP-2 concentrations were associated with very high levels of translocation (Fig 4B). Other biomarkers (CRP, sCD14, CD163, LBP) were not associated with LPS, either in the whole group or stratified by HIV.

We modelled the relationship between LPS and morphometric measures separately in a multivariable fractional polynomial regression model, and LPS was associated only with villus epithelial perimeter (β = 10136; *P* = 0.01) and villus SA:volume ratio (β = 59006; *P* = 0.007). The relationship between LPS and villus SA:volume ratio was U-shaped and significant in a fractional polynomial regression model (Fig 4C). Counter-intuitively, across most of the range of villus surface area analysed, greater surface area was associated with increased translocation, and if a linear regression is used the association is still positive overall (ρ = 0.46; *P* = 0.003), consistent with greater surface area leading to greater levels of translocation.

### Zinc uptake testing

Baseline plasma zinc concentrations were low, with median just above the lower limit of normal concentration (Table 2), and 21/48 (44%) participants had plasma concentrations below 11 μmol/l which are consistent with deficiency. The median increment in plasma zinc at 3 hours was 6.3 μmol/l, with 30/47 (64%) having an attenuated rise (compared to ref 28) indicative of impaired net uptake. HIV infection was associated with higher risk of attenuated uptake over 3 hours (OR 1.6, 95%CI 1.1,2.3, *P* = 0.05), but baseline zinc concentration did not differ with HIV status. In multivariate analysis using all the CLE and potential determinants used in the translocation regression models, zinc uptake was associated negatively with smoking (β = -11.7; *P* = 0.02) and positively with GLP-2 (β = 2.70; *P* = 0.03) and baseline plasma zinc concentration (β = 0.616; *P* = 0.006)(Fig E in S1 Text). We conclude that higher zinc uptake was associated with higher circulating levels of a hormone which increases epithelial cell mass and therefore absorptive area.

### Transcriptome analysis

We sequenced RNA from 4 participants with very low or undetectable plumes and 4 with frequent plumes (Table C in S1 Text). RNA sequencing yielded between 7.35 and 10.22 million reads per sample of 2μg RNA, with unique matches to 20,001 gene transcripts (between 98.2% and 99.1% mapped to genome). Agnostic analysis of the transcriptome data from 4 participants with mild and 4 participants with very severe fluorescein leak (defined by frequency of plumes) yielded 23 differentially expressed genes (Table 3). These included down-regulated transcripts involved in metal ion uptake (intelectin, lipocalin, DMT-1, and lactotransferrin), anti-protease activity (SPINK-4, serpin-9), mucosal protection (trefoil factor 3), and host defence (human defensins 5 and 6, Reg-3α), and up-regulated transcripts involved in inflammation (c-fos, fos B, granulysin, HLA-DR-β5 and CXC10). Trefoil factor 3 immunostaining (Fig F in S1 Text) and counting of goblet cells showed that reduced mRNA was not due to loss of goblet cells.

**Table 3:** Differentially expressed genes in 4 biopsies from duodenum with multiple plumes seen by confocal laser endomicroscopy compared to 4 with minimal plumes.

Gene ID	Gene name/function	Functional group	Locus	log2Ratio	Fold change	Probability score
1671	Human defensin 6	Paneth cell antimicrobial peptides	479	-2.17316	0.222	0.826343
1670	Human defensin 5		449	-1.92632	0.264	0.813815
5068	Reg 3α		1117	-1.87921	0.272	0.80937
7033	Trefoil factor 3; epithelial repair peptide	Epithelial repair peptide	1054	-2.16378	0.224	0.820769
327657	Serpin 9; protease inhibitor	Anti-proteolytic	1851	-12.8255	0.00014	0.857051
27290	SPINK 4; protease inhibitor		386	-4.41835	0.047	0.872211
3934	Lipocalin 2; iron sequestration	Cation uptake and sequestration from pathogens	840	-2.18948	0.219	0.81402
4057	Lactotransferrin; antimicrobial peptide and iron sequestration		2593	-5.38692	0.024	0.80093
4891	Solute carrier family 11, member 2 (DMT-1); iron, manganese and zinc uptake		4424	-1.84634	0.277	0.804259
55600	Intelectin 1; lactoferrin receptor		386	-3.6465	0.080	0.834541
1179	Chloride channel accessory 1	Nutrient and electrolyte transport, not cations	3123	-3.4113	0.094	0.827942
29802	Pre-B lymphocyte 3	Immune function and inflammation	602	-4.7429	0.037	0.815015
2353	c-fos; AP-1 subunit		2158	2.765478	6.80	0.854176
2354	fos B; AP-1 subunit		3776	7.030998	130.8	0.892508
10578	granulysin; cytotoxic cell effector molecule		995	2.736818	6.67	0.80525
3127	HLA-DR β5; antigen presentation		1171	2.412484	5.32	0.842403
3627	chemokine C-X-C ligand 10; chemokine, interferon-γ pathway		1227	2.902382	7.48	0.807325
1543	Cytochrome P450, family 1, subfamily A, no 1	Xenobiotic metabolism	2608	2.364329	5.15	0.832779
139728	CaM kinase; PNCK	Role or function unclear in this context	1888	-3.69906	0.077	0.805404
1843	Dual specificity phosphatase 1		2040	1.946366	3.88	0.806404
692203	Small nucleolar RNA, C/Dbox 88B		97	-12.4208	0.00018	0.820723
100861532	unknown function		13357	-3.20998	0.108	0.822118
260436	chromosome 4, ORF7		573	-7.68911	0.0048	0.937805

The **Probability** shown is derived from RKPM-normalised values using NOIseq (non-parametric) computation of signal to noise ratio, and using a standard threshold, q, of 0.8 which corresponds to an odds ratio of 4:1 that the gene is differentially expressed. Values of Probability shown indicate the probability that a given transcript is differentially expressed; higher values indicate greater probability of differential expression; values greater than 0.8 are accepted as significant [29].

## Discussion

Environmental enteropathy has emerged as an important problem in global health, particularly in children. Improved child growth and development, reduced burden of vaccine-preventable diseases, and improved micronutrient status are three of the possible rewards for a successful approach to its prevention [33]. The data we present here from Africans living in Africa suggest that the small intestinal lesion, which is ubiquitous, can be remarkably severe, even in apparently healthy adults with no symptoms of disease and without recent exposure to noxa known to cause specific enteropathies such as helminth infections or NSAIDs. Average villus height was markedly reduced against European normal values. The confocal endomicroscopy images in many cases show free leakage of fluorescein from systemic circulation to gut lumen, and animal work has shown that when this efflux is visible, a bidirectional flux is actually in process allowing the ingress of bacteria, antigens and toxins into the body [24]. Immunolocalisation of claudin 4 delineates severe lesions, particularly at the villus tip, just where endomicroscopy shows leakage to be frequent. Funnels of tight junction proteins, such as claudin 4, characterise points of increased cell shedding and epithelial separation, which were seen prominently in these biopsies. We show that fluorescein leak due to epithelial breaches was associated with high plasma lipopolysaccharide, a component of Gram negative cell walls. This study was restricted to adults, and it suggests that many Zambian adults are living with a constant source of chronic inflammation. We think it likely, though it is not proved, that this has adverse consequences for health in the long term. A parallel study in children is under way.

There are obvious limitations to this study, notably that it is purely cross-sectional and the sample size is limited by the intensive and time-consuming nature of the procedures undertaken in an African setting where environmental enteropathy is the norm. Furthermore, the measurement of translocated bacterial components is largely dependent on one assay, LPS. However, confocal laser endomicroscopy has made possible an understanding of the severity of epithelial disruption, as the large image sample size (between 35 and 225 images per procedure) and the fact that the epithelium is imaged *in vivo* give considerable confidence to our assessment that many of the defects seen in histological images are not artefactual. The claudin 4 funnels at the villus tip are an important proxy for cell shedding as we have previously shown that tight junction proteins redistribute around epithelial cells during shedding [32,34]. The increased funnel formation shows that the epithelial damage is a direct consequence of increased shedding. It is already known the LPS and TNF are potent stimuli for cell shedding [23,35], although in EE it is unclear whether this is due to increased apoptosis (which occurs physiologically at the villus tip), necroptosis, or necrosis. It is therefore plausible that initial minor defects in the barrier lead to the ingress of bacteria increasing tissue concentrations of TNF, increasing pathology in a positive feedback loop.

We found evidence of a U-shaped relationship between translocation and villus surface area. This observation suggests that the reduced villus surface area in enteropathic disorders may act at least in part to mitigate the translocation burden in the presence (as in this population) of ubiquitous epithelial defects. Thus, a reduced epithelial surface area may confer a survival advantage in the presence of heavy exposure to environmental noxa. The selection pressure could be high, as the circulating concentrations of LPS in our healthy volunteers was two orders of magnitude higher than in Italian children with fatty liver disease [15]. This finding needs to be confirmed in future work.

HIV does not seem to add to the epithelial damage of EE in a population such as this where both frequently co-exist, with the exception of villus surface area. This is in contrast to work in the USA and elsewhere which suggests that HIV may predispose to bacterial translocation [10,36]. In a previous study of morphometry over time [20] we found that HIV had little effect on mucosal architecture over and above EE, except for an increase in crypt depth at all stages of infection and an increase in lactulose permeation only in late stage HIV (CD4 cells < 200 cells/μl). In this study we also found reduced epithelial surface area in HIV, but no change in translocation. However, there was a striking increase in CRP in HIV infected participants which cannot be explained by bacterial translocation and was not accompanied by changes in other monocyte-derived molecules such as sCD14 or CD163. Our data do not completely fit with a simple model in which HIV drives bacterial translocation leading to systemic immune activation [10], and other processes undoubtedly contribute more in Africa than in temperate climates. Furthermore, the majority of studies conducted using morphometry or lactulose permeation indicate that increased leakiness of the small intestine is a hallmark of advanced HIV disease [3], whereas our participants had early or well-controlled disease. At least in a population with a good HIV treatment programme, the presence of EE is the primary driver of translocation and inflammation, and HIV has only a modest additional impact.

The zinc challenge test is a crude assessment of the net ability of the small intestine to take up zinc from a test dose, and cannot replace isotopic analysis of absorption and losses from endogenous zinc [37]. However, overall uptake of zinc from ingested zinc is important for human health, and here we show that it is attenuated in a large proportion of people with EE. Baseline zinc in plasma was also low, which probably indicates subclinical zinc deficiency. We were unable to correlate zinc uptake to epithelial surface area measured morphometrically, but in our transcriptomic analysis we found that more severe enteropathy was associated with reduced expression of DMT-1 which may contribute to uptake of zinc [38], especially supra-physiological doses. Hypochlorhydria was common in our patients, as we have previously reported [39], but is unlikely to be the explanation for impaired zinc uptake which does not depend on gastric acid [40].

GLP-2 is a major driver of intestinal adaptation [41,42] and a long-acting analogue, teduglutide, has been shown in clinical trials to augment intestinal adaptation in patients with short bowel [43]. GLP-2 is secreted by L cells of the distal gut, probably in response to nutrients that have not been absorbed proximally. Here we found clear evidence that low circulating GLP-2 concentrations were associated with very high levels of translocation. The absolute concentration of GLP-2 is not easy to establish as different techniques (ELISA, RadioImmunoAssay) have different sensitivities and give different results. Average normal circulating levels have variously been reported as 0.1 ng/ml [44] or 42pg/ml by RIA [45]_,_ and 0.4ng/ml [46] or 30pg/ml by ELISA [47], and importantly the manufacturers of the ELISA we used report normal concentrations with a lower limit of 3.6 ng/ml, so overall the concentrations in our participants were probably low. Our findings would be consistent with a primary endocrine failure of GLP-2 synthesis rather than a failure of end-organ responsiveness (which would be expected to result in elevated GLP-2 levels).

Hypothesis-free analysis of the transcriptome, comparing biopsies from severe and very mild enteropathy, revealed some important new insights. We have previously shown that the Paneth cell antimicrobials HD5 and HD6 are down-regulated in EE [48], and these data confirm our earlier findings. The other transcriptional changes require further work, but are consistent with findings in other inflammatory conditions and with our data on T cell activation in EE[49]. The down-regulation of DMT-1 might help explain the reduced zinc uptake. Although the role played by DMT-1 in zinc transport is less secure than its role in iron (II) and manganese uptake [38], it seems likely to play a role in uptake of supra-physiological doses such as the 25mg dose we administered, and which are used in oral rehydration therapies for infectious diarrhoea and in micronutrient supplementation. Three other genes involved in iron uptake were also down-regulated. The down-regulation of antiprotease activity may help explain the epithelial defects which we have found (Figs 2 and 3) to be correlated with translocation as they would render the mucosa susceptible to matrix degradation. SPINK-4 has also been implicated in coeliac disease [50]. Trefoil factor 3 is a mucosal protective peptide, secreted by goblet cells into the gut lumen and a major driver of epithelial repair [51,52]. Down-regulation of TFF3 synthesis in severe EE may imply a failure of a protective mechanism. In the light of our evidence that low circulating GLP-2 is associated with increased translocation we postulate that EE is driven or perpetuated by a failure of homeostatic repair mechanisms.

In conclusion, we have shown for what we believe to be the first time a significant epithelial barrier defect in EE *in vivo*, which provides a plausible pathway for the bacterial translocation observed in many populations. The epithelial damage, in part at least, seems to follow from a failure of mucosal maintenance systems. Our data suggest that reduced mucosal defence peptides, including antimicrobial peptides HD5 and 6, reduced circulating hormones such as GLP-2, and reduced locally secreted factors such as trefoil factor 3 and anti-proteases may contribute to the translocation and malabsorption seen in severe enteropathy. In the light of these data, clinical trials of sources of these factors may be considered.

## Supporting Information

S1 Text **Supplementary information is included on methodological details: quantitative analysis of images from confocal laser endomicroscopy, together with Table A, which demonstrates correlations between confocal measurements; morphometric analysis and a comparison with published values of three morphometric parameters (Table B); claudin 4 immunostaining; measurement of blood and stool markers; and RNA sequencing and analysis, including baseline data on the subgroup in which sequencing was carried out (Table C).** Seven supplementary Figures are included. **Fig A** Morphometric assessment. Villus height (VH) is measured at 147 μm, Crypt depth (CD) at 139 μm, and Villus width at 147 μm. Villus perimeter is measured at 785 μm, representing epithelial surface area when the horizontal edge is subtracted, and villus cross-sectional area at 0.0181μm^2^, representing villus volume. For all morphometry, it is essential that crypts are seen throughout their length so that correct orientation along the crypt-villus axis is confirmed. **Fig B** shows correlations between fluorescein leak and (A) erosions, and (B) cell shedding events. **Fig C** shows the correlation between FABP and plumes. **Fig D** Further images of epithelial breaches identified by confocal laser endomicroscopy (A,D), claudin 4 immunostaining (B,E,G,J) and histology (C,F,H) from three individuals (A-C, D-F and G-H). No corresponding endomicroscopy image could be found in the stack of images from the third participant (G,H). In A, a plume (thick arrow) is associated with a microerosion (thin arrow), and in D a microerosion is shown (arrow). In B and E, claudin 4 immunostaining outlines early epithelial separation (arrows), also seen in C and F (arrows). In G and H epithelial separation has progressed to the point where basement membrane is exposed (arrows). In K, claudin 4 immunoreactivity, from a participant with very mild enteropathy, is shown in a more normal distribution, with points of reactivity near the luminal end of the lateral intercellular space corresponding to the expected position of tight junctions (small white arrows), and some basolateral staining near the villus tip at another point of cell shedding (green arrow). **Fig E** Scatter plot of log-transformed change in zinc uptake and GLP-2 concentration (β = 0.12; *P* = 0.02 in the multivariate model). **Fig F** TFF3 immunostaining showing good immunoreactivity in goblet cells. **Fig G** Normal images of duodenum using confocal laser endomicroscopy.
(DOCX)
