# Correction: Endomicroscopic and Transcriptomic Analysis of Impaired Barrier Function and Malabsorption in Environmental Enteropathy

**DOI:** 10.1371/journal.pntd.0010505

**Published:** 2022-06-02

**Authors:** Paul Kelly, Ellen Besa, Kanekwa Zyambo, John Louis-Auguste, James Lees, Themba Banda, Rose Soko, Rosemary Banda, Beatrice Amadi, Alastair Watson

There is an error in Table 3. The sign of the fold expression is reversed and the log2Ratio is inverted. The errors arose through mixing up group A and B.

**Table 3:** Differentially expressed genes in 4 biopsies from duodenum with multiple plumes seen by confocal laser endomicroscopy compared to 4 with minimal plumes.

Gene ID	Gene name/function	Functional group	Locus	log2Ratio	Fold change	Probability score
1671	Human defensin 6	Paneth cell antimicrobial peptides	479	2.17316	4.505	0.826343
1670	Human defensin 5		449	1.92632	3.788	0.813815
5068	Reg 3α		1117	1.87921	3.676	0.80937
7033	Trefoil factor 3; epithelial repair peptide	Epithelial repair peptide	1054	2.16378	4.464	0.820769
327657	Serpin 9; protease inhibitor	Anti-proteolytic	1851	12.8255	7143	0.857051
27290	SPINK 4; protease inhibitor		386	4.41835	21.28	0.872211
3934	Lipocalin 2; iron sequestration	Cation uptake and sequestration from pathogens	840	2.18948	4.566	0.81402
4057	Lactotransferrin; antimicrobial peptide and iron sequestration		2593	5.38692	41.67	0.80093
4891	Solute carrier family 11, member 2 (DMT-1); iron, manganese and zinc uptake		4424	1.84634	3.610	0.804259
55600	Intelectin 1; lactoferrin receptor		386	3.6465	12.5	0.834541
1179	Chloride channel accessory 1	Nutrient and electrolyte transport, not cations	3123	3.4113	10.64	0.827942
29802	Pre-B lymphocyte 3	Immune function and inflammation	602	4.7429	27.03	0.815015
2353	c-fos; AP-1 subunit		2158	-2.765478	0.147	0.854176
2354	fos B; AP-1 subunit		3776	-7.030998	0.008	0.892508
10578	granulysin; cytotoxic cell effector molecule		995	-2.736818	0.150	0.80525
3127	HLA-DR β5; antigen presentation		1171	-2.412484	0.188	0.842403
3627	chemokine C-X-C ligand 10; chemokine, interferon-γ pathway		1227	-2.902382	0.134	0.807325
1543	Cytochrome P450, family 1, subfamily A, no 1	Xenobiotic metabolism	2608	-2.364329	0.194	0.832779
139728	CaM kinase; PNCK	Role or function unclear in this context	1888	3.69906	12.99	0.805404
1843	Dual specificity phosphatase 1		2040	-1.946366	0.258	0.806404
692203	Small nucleolar RNA, C/Dbox 88B		97	12.4208	5555.6	0.820723
100861532	unknown function		13357	3.20998	9.259	0.822118
260436	chromosome 4, ORF7		573	7.68911	208.3	0.937805

The **Probability** shown is derived from RKPM-normalised values using NOIseq (non-parametric) computation of signal to noise ratio, and using a standard threshold, q, of 0.8 which corresponds to an odds ratio of 4:1 that the gene is differentially expressed. Values of Probability shown indicate the probability that a given transcript is differentially expressed; higher values indicate greater probability of differential expression; values greater than 0.8 are accepted as significant [29].
