# Supplementary material for: Endomicroscopic and Transcriptomic Analysis of Impaired Barrier Function and Malabsorption in Environmental Enteropathy
Source: PLoS Negl Trop Dis. 2016 Apr 6;10(4):e0004600. doi: 10.1371/journal.pntd.0004600 (PMC4822862; doi:10.1371/journal.pntd.0004600)

**Supplementary material**

*Morphometry*

Measurements of villus height (VH), crypt depth (CD), and villus width (VW) were made using linear measurements (Figure S2). Measurements of villus perimeter and cross-sectional area were made by drawing the regional boundary (Fig A).


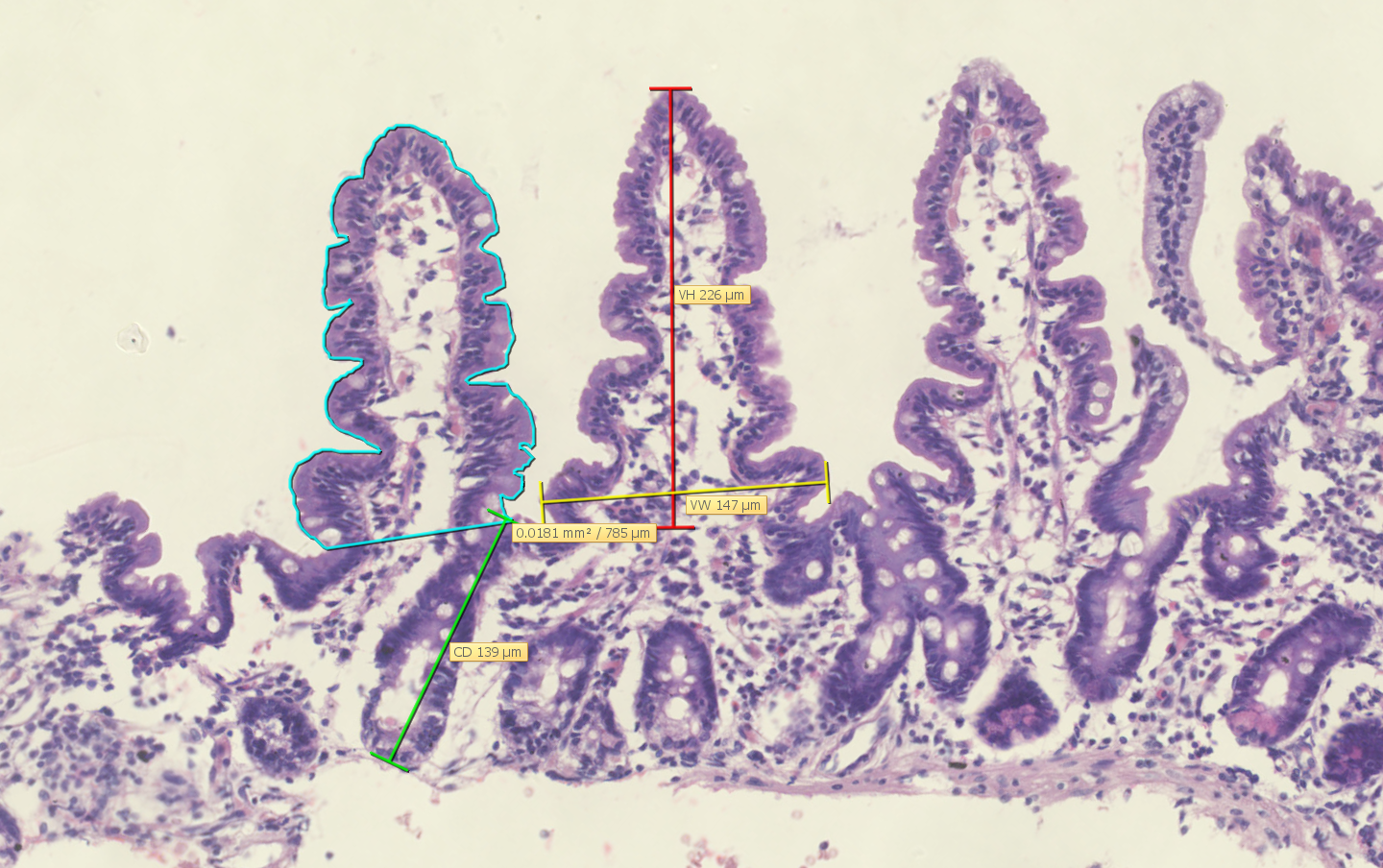


**Figure A** Morphometric assessment. Villus height (VH) is measured at 147 μm, Crypt depth (CD) at 139 μm, and Villus width at 147 μm. Villus perimeter is measured at 785 μm, representing epithelial surface area when the horizontal edge is subtracted, and villus cross-sectional area at 0.0181μm^2^, representing villus volume. For all morphometry, it is essential that crypts are seen throughout their length so that correct orientation along the crypt-villus axis is confirmed.

*Quantitative analysis of images from confocal laser endomicroscopy*

A quantitative image analysis protocol was adopted. This protocol was applied systematically so as to obtain a series of representative images of the epithelial cells in that individual. The median number of images collected was 381 (range 111-569). In the first pass through the stack, unusable images (usually due to motion artefact) were discarded, leaving a median of 167 (range 69-290) images. In the second pass, duplicate images were discarded leaving a set (median 134, range 35-225) of unique representative images.

Fluorescein leak was measured as the number of images in which the duodenal lumen was observed to be as bright or brighter than the tissue (Fig 1C), and expressed as a proportion of the total number of images being assessed. The number of plumes (focal leaks of fluorescein, Fig 1B), the number of single epithelial defects (Fig 1D), the number of microerosions (Fig 1E) and the number of images with complete loss of integrity (Fig 1F) were all counted in sequential passes through the stack of images, and expressed as a proportion of the number of images analysed. These measurements were highly correlated (Table A, Figure B).

To estimate inter-observer variation, a full range of quantitative measurements set out in Table S1 were made by two independent observers (JLA and PK) in 39 image sets and the results compared. Strong agreement was obtained for fluorescein leakage (ρ = 0.94; *P*<0.0001), plumes (ρ = 0.83; *P*<0.0001) and silhouettes (ρ = 0.80; P<0.0001), but weaker for cellular lesions such as apoptosis (ρ=0.40; *P* = 0.01) or single cell defects (ρ=0.41; *P* = 0.01). Erosions were intermediate (ρ=0.46; *P*=0.004). In both cases where Watson score was 1, there was agreement between observers.

**Table A** Correlations between confocal measurements

| Spearman’s ρ  (n   | Fluorescein leak | Plumes | Single defects | Erosions | Apoptotic bodies | Shedding | Breakdown | Silhouettes |
| --- | --- | --- | --- | --- | --- | --- | --- | --- |
| Fluorescein  leak into lumen | 1 |  |  |  |  |  |  |  |
| Plumes | 0.606 | 1 |  |  |  |  |  |  |
| Single cell defects | 0.644 | 0.717 | 1 |  |  |  |  |  |
| Microerosions | 0.724 | 0.764 | 0.863 | 1 |  |  |  |  |
| Apoptotic bodies | 0.548 | 0.582 | 0.681 | 0.642 | 1 |  |  |  |
| Cell shedding events | 0.874 | 0.671 | 0.698 | 0.819 | 0.549 | 1 |  |  |
| Breakdown | 0.645 | 0.353 | 0.399 | 0.528 | 0.389 | 0.598 | 1 |  |
| Silhouettes | 0.567 | 0.294 | ns | 0.289 | ns | 0.509 | 0.365 | 1 |

ns, not significantly correlated (*P* > 0.05)

**Figure B** Correlations between fluorescein leak and (A) erosions, or (B) cell shedding events

A

B

**Figure C** Correlation between FABP and plumes

**Table B** Normal mucosal morphological measurements, drawn from two papers.

|  | Lipski et al, adults,  med (IQR) | Penna et al, adults,  mean (SD) | Penna et al,  children,  mean (SD) | P (for the difference between adults and children in Penna study |
| --- | --- | --- | --- | --- |
| Villus height (μm) | 487  (422-542) | 368 (59) | 332 (45) | 0.04 |
| Crypt depth (μm) | 161  (136-197) | 119 (18) | 169 (28) | <0.0001 |
| Villus:crypt ratio | 3.2  (3.1-3.5) | 3.17 (0.8) | 2.00 (0.35) | <0.0001 |

Lipski PS, Bennett MK, Kelly PJ, James OFW. Ageing and duodenal morphometry. J Clin Path 1992; 45: 450-452. Penna FJ, Hill ID, Kingston D, Robertson K, Slavin G, Shiner M. Jejunal mucosal morphometry in children with and without gut symptoms and in normal adults. J Clin Path 1981; 34: 386-392.

*Claudin 4 immunostaining*

Claudin 4 immunostaining was performed on paraffin-embedded formalin-fixed biopsy tissue using a rabbit polyclonal antibody to human claudin 4 (ab53156, Abcam, Cambridge, UK) at 1/100 dilution, following antigen retrieval (water bath, 10 minutes, pH 9) and imaged using a Cy3-conjugated goat anti-rabbit secondary (ab97075, Abcam) under a confocal microscope (Zeiss LSM710). Quantification was performed using ImageJ software to compare relative staining intensity between biopsies. Scoring of villus tip breakdown and epithelial breaks was carried out on claudin 4-stained sections by an independent observer (JL), and scored as present or absent.

*Measurement of blood and stool markers*

Quantification of LPS was by the Limulus Amoebocyte Lysate (LAL) assay (Associates of Cape Cod, Liverpool, UK). LPS binding protein, CRP, sCD14, and CD163 were measured by ELISA (R&D systems, Abingdon, UK). Total immunoreactive GLP-2 was measured in serum by ELISA (Millipore, St Charles, MO), which has a lower limit of detection of 0.3 ng/ml. Intestinal FABP was measured by ELISA (Hycult, Plymouth Meeting, PA). Faecal α1-antitrypsin was measured by ELISA (Immuchrom, Oxford Biosystems, Oxford, UK.

*RNA sequencing*

Biopsies for RNA sequencing, which had been snap-frozen in liquid nitrogen and stored at -80^o^C, were retrieved and RNA extracted using Trizol (Ambion, Life Technologies, Paisley, UK) and RNAeasy columns (Qiagen, Manchester, UK) prior to shipping to the Beijing Genomics Institute on dry ice. Reverse transcription was carried out following mRNA enrichment on poly-A conjugated magnetic beads, and libraries constructed for sequencing using Ion Proton. Three biological replicates of each sample were sequenced. The reference sequences used in this study were the human genome and transcriptome sequences downloaded from the UCSC website (<http://genome.ucsc.edu/index.html>, version hg19). After the removal of low quality reads, clean reads were aligned to the reference genome or transcriptome using TMAP software. No more than five mismatches were allowed in the alignment of each read. Differentially expressed genes were identified in RPKM-normalised databases using NOIseq analysis^29^, with a cut-off Probability > 0.8. Basic description of the transcriptomic dataset is reported in conformity with guidelines for RNAseq (ENCODE, v 1.0, downloaded from [www.genome.ucsc.edu](http://www.genome.ucsc.edu) on 5^th^ July, 2015).

**
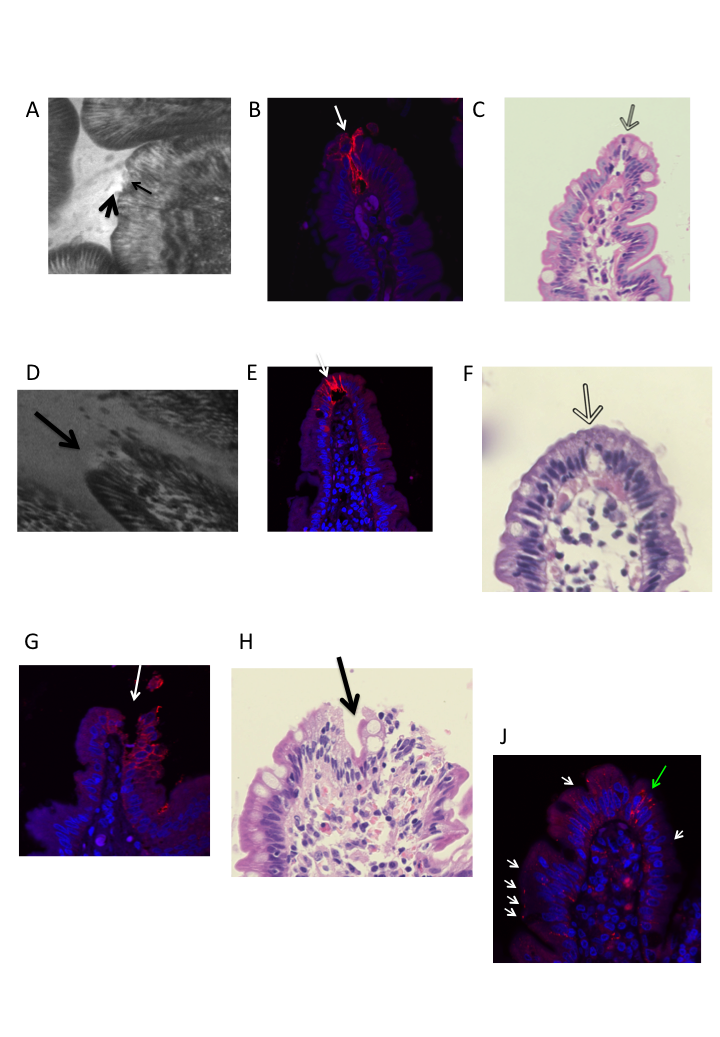
**

**Figure D** Further images of epithelial breaches identified by confocal laser endomicroscopy (A,D), claudin 4 immunostaining (B,E,G,J) and histology (C,F,H) from three individuals (A-C, D-F and G-H). No corresponding endomicroscopy image could be found in the stack of images from the third participant (G,H). In A, a plume (thick arrow) is associated with a microerosion (thin arrow), and in D a microerosion is shown (arrow). In B and E, claudin 4 immunostaining outlines early epithelial separation (arrows), also seen in C and F (arrows). In G and H epithelial separation has progressed to the point where basement membrane is exposed (arrows). In K, claudin 4 immunoreactivity, from a participant with very mild enteropathy, is shown in a more normal distribution, with points of reactivity near the luminal end of the lateral intercellular space corresponding to the expected position of tight junctions (small white arrows), and some basolateral staining near the villus tip at another point of cell shedding (green arrow).

**Figure E** Scatter plot of log-transformed change in zinc uptake and GLP-2 concentration (β = 0.12; *P* = 0.02 in the multivariate model).

**
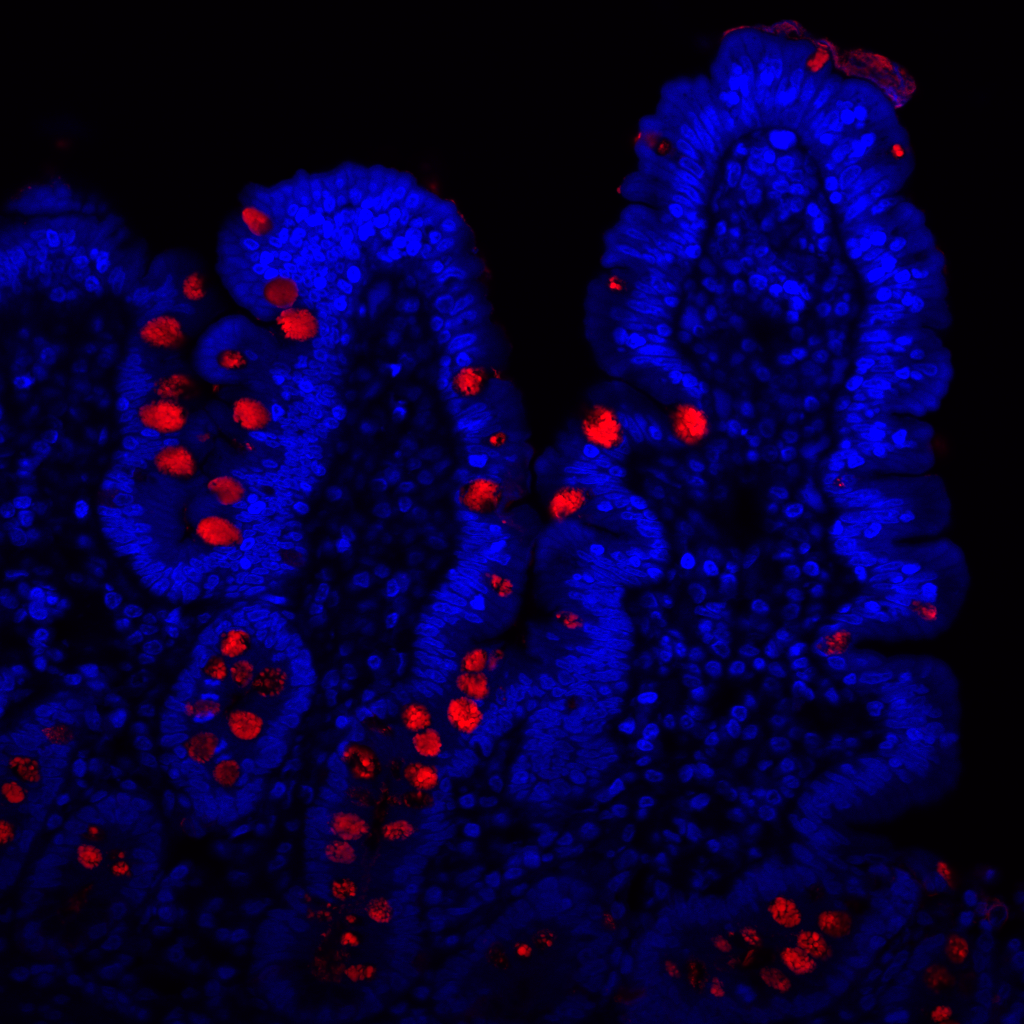
**

**Figure F** TFF3 immunostaining showing good immunoreactivity in goblet cells

**Table C** Clinical characteristics of participants whose biopsies were used for RNA sequencing

|  | Low plumes | High plumes | P |
| --- | --- | --- | --- |
| Sex (M:F) | 1:3 | 1:3 | 1.00 |
| Age (years; median, IQR) | 39 (26-50) | 25 (22-29) | 0.19 |
| HIV seropositive | 1 of 4 | 0 of 4 |  |
| VH (μm; median, IQR) | 200 (196-259) | 223 (201-250) | 0.72 |
| CD (μm; median, IQR) | 161 (142-187) | 169 (167-172) | 0.48 |
| BMI (kg/m^2^; median, IQR) | 23.0 (18.1-32.1) | 21.7 (20.1-23.6) | 1.00 |
| MUAC (cm; median, IQR) | 27.5 (23.3-32.5) | 27.5 (24.7-28.5) | 0.77 |

**Figure G** Normal images of duodenum using confocal laser endomicroscopy


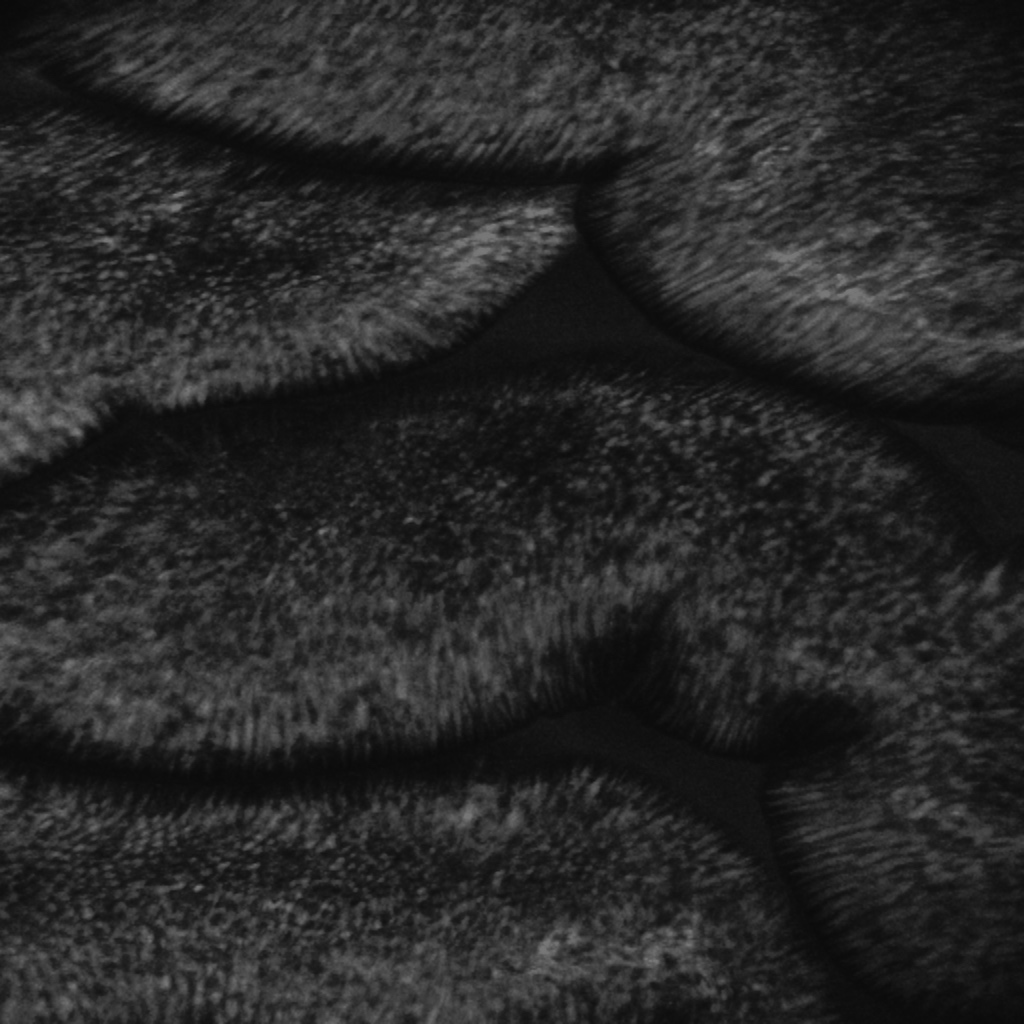


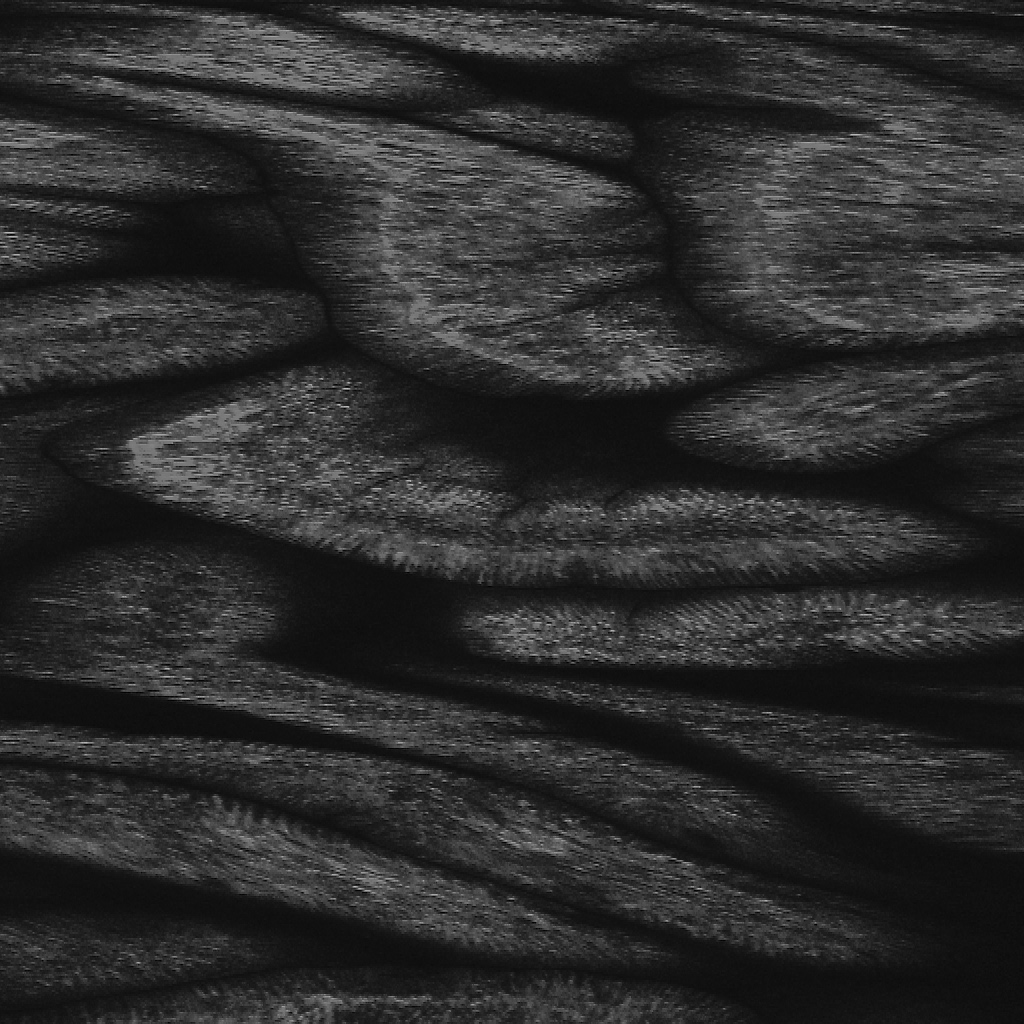


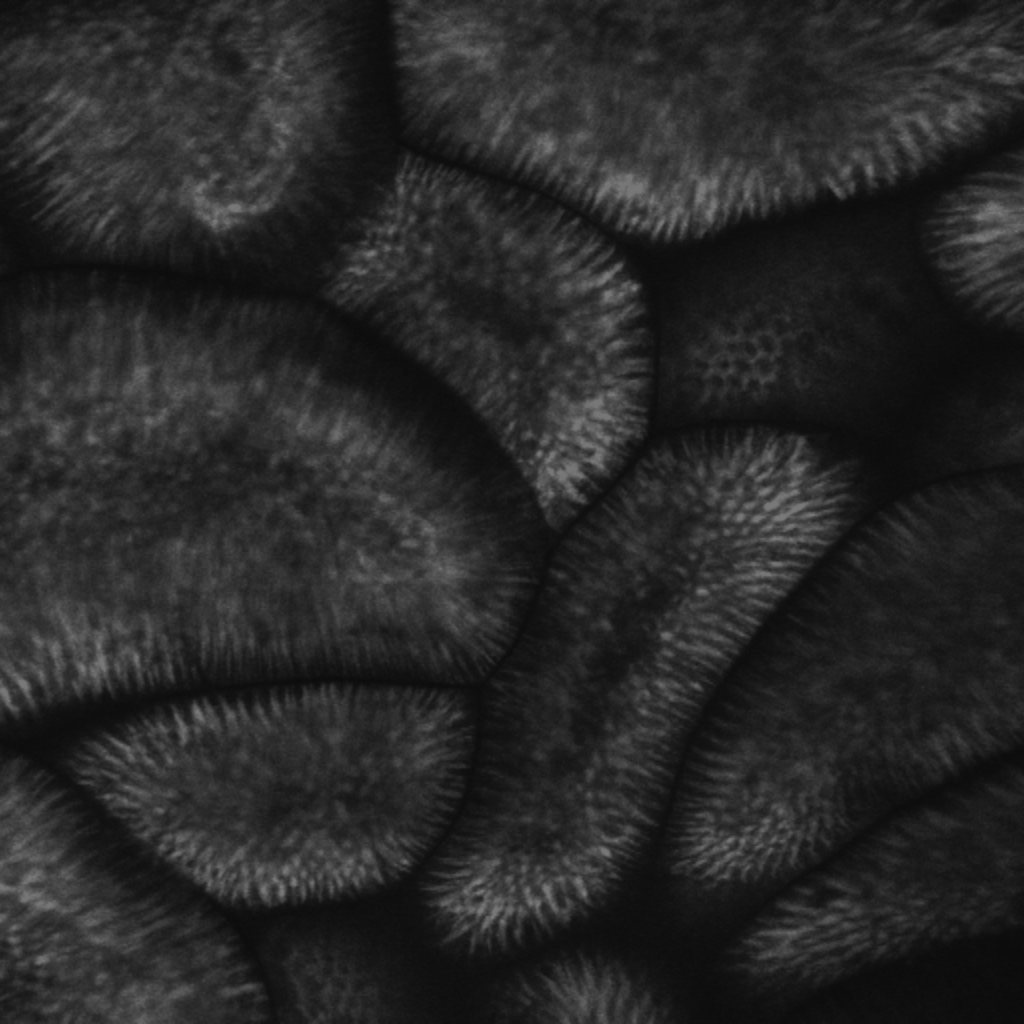

Supplement: S1 Text — Supplementary information is included on methodological details: quantitative analysis of images from confocal laser endomicroscopy, together with Table A, which demonstrates correlations between confocal measurements; morphometric analysis and a comparison with published values of three morphometric parameters (Table B); claudin 4 immunostaining; measurement of blood and stool markers; and RNA sequencing and analysis, including baseline data on the subgroup in which sequencing was carried out (Table C). Seven supplementary Figures are included. Fig A Morphometric assessment. Villus height (VH) is measured at 147 μm, Crypt depth (CD) at 139 μm, and Villus width at 147 μm. Villus perimeter is measured at 785 μm, representing epithelial surface area when the horizontal edge is subtracted, and villus cross-sectional area at 0.0181μm2, representing villus volume. For all morphometry, it is essential that crypts are seen throughout their length so that correct orientation along the crypt-villus axis is confirmed. Fig B shows correlations between fluorescein leak and (A) erosions, and (B) cell shedding events. Fig C shows the correlation between FABP and plumes. Fig D Further images of epithelial breaches identified by confocal laser endomicroscopy (A,D), claudin 4 immunostaining (B,E,G,J) and histology (C,F,H) from three individuals (A-C, D-F and G-H). No corresponding endomicroscopy image could be found in the stack of images from the third participant (G,H). In A, a plume (thick arrow) is associated with a microerosion (thin arrow), and in D a microerosion is shown (arrow). In B and E, claudin 4 immunostaining outlines early epithelial separation (arrows), also seen in C and F (arrows). In G and H epithelial separation has progressed to the point where basement membrane is exposed (arrows). In K, claudin 4 immunoreactivity, from a participant with very mild enteropathy, is shown in a more normal distribution, with points of reactivity near the luminal end of the latera [file pntd.0004600.s001.docx]
